# Clinical outcomes following ankle fracture: a cross-sectional observational study

**DOI:** 10.1186/s13047-014-0050-9

**Published:** 2014-11-28

**Authors:** Ganit Segal, Avi Elbaz, Alon Parsi, Ziv Heller, Ezequiel Palmanovich, Meir Nyska, Zeev Feldbrin, Benjamin Kish

**Affiliations:** AposTherapy Research Group, 1 Abba Even Blvd, Herzliya, 46733 Israel; Department of Orthopedic Surgery, Meir Medical Center, Kfar-Saba, Israel; Department of Orthopaedic Surgery, Wolfson Medical center, Holon, Israel, affiliated to the Sacker Faculty of Medicine, Tel Aviv University, Tel Aviv, Israel

**Keywords:** Ankle fracture severity, Gait, Clinical outcomes

## Abstract

**Background:**

The purpose of the current study was to examine objective and subjective differences between three severity groups of ankle fractures patients compared to healthy controls.

**Methods:**

This was a case-controlled study. 92 patients with an ankle fracture injury of which 41 patients were eligible to participate in the study. 72 healthy people served as controls. All patients underwent a computerized gait test, completed self-assessment questionnaires (The Foot and Ankle Outcome Score (FAOS) and the SF-36), evaluated with the American Foot and Ankle Score (AOFAS) form and completed the 6-min walk test. The control group performed a computerized gait test and completed the SF-36 health survey.

**Results:**

All ankle fracture patients presented compromised gait patterns and limb symmetry compared to controls (*p* < 0.05). Ankle fracture patients also had lower SF-36 scores compared to controls (*p* < 0.05). Significant differences were found between the unimalleolar group compared to the bimalleolar and trimalleolar groups in most parameters, except for the FAOS scores. There were no significant differences between the bimalleolar fracture group and the trimalleolar fracture groups.

**Conclusions:**

Although all fracture severity classification groups presented a compromised gait pattern and worse clinical symptoms compared to controls, it seems that patients with a unimalleolar fracture is a different group compared to bimalleolar and trimalleolar fracture. Furthermore, it seems that bimalleolar fracture and trimalleolar fracture affect the gait pattern and clinical symptoms to an equal extent, at least in the short-term.

**Trial registration:**

NCT01127776.

## Background

Ankle fractures are one of the most common injuries of the lower limb [1]. There has been a constant increase in ankle fracture rates amongst young, active patients as well as in the elderly population over the last several decades [2,3]. Operative treatment of ankle fracture includes open reduction and internal fixation [4], followed by immobilization and rehabilitation [5–7].

Ankle fractures severity can be defined and classified to three sub-groups including unimalleolar, bimalleolar and trimalleolar fractures. Several studies have examined the differences between severity groups in regard to functional outcomes and showed conflicting results. Some concluded that a fracture severity classification is a consistent predictor of functional outcome following surgery [8–10]. However, recent work by Egol et al. concluded that the type of fracture had no influence on functional recovery [11]. Most studies used self-assessment questionnaires and functional scores to evaluate the functional status of the patient post an ankle fracture surgery. Although questionnaires are considered a valid method of assessment, they are subjective, and objective methods of evaluation are warranted.

Gait analysis is widely used to characterize functional performance of different populations [12–15]. It is also used as an outcome measure for decision making and for evaluating different treatments [16–19]. Recently, functional severity classification for patients with knee osteoarthritis, which is based on gait analysis, was presented [20]. To the best of our knowledge, there is limited information regarding the changes in gait patterns following ankle fracture. We found one study by Becker et al. who evaluated gait symmetry post ankle fracture surgery. They concluded that after 18 months of surgery, gait symmetry in plantar pressure distribution was achieved. They did not find a difference between fracture severity groups [16]. Their study was done on a young population and months post-surgery. The purpose of the current study was to examine objective (gait analysis) and subjective (pain, function and quality of life) differences between three severity groups of ankle fractures patients immediately with weight-bearing allowance compared to healthy controls.

## Methods

This study is part of a wider, double blind, randomized controlled trial examining the effect of a new rehabilitation intervention for patients following ankle fracture. Ethic approval was obtained from by the Helsinki Committee of the participating medical center. The trial registration number is NCT01127776.

### Patients

Ninety-two patients with acute ankle fracture were referred to the study between December 2010 and August 2013. All patients were treated operatively with open reduction and internal fixation according to AO/ASIF methods [21], and were instructed to avoid weight-bearing for 6 weeks. Patients who had a syndesmosis injury were treated with a 3.5 mm 4 cortex screw, which was removed 3 months post-surgery. Patients were recruited to the study during their follow-up examination with the orthopedic surgeon, who offered them to join the study. Patients were contacted by the research team, and once weight-bearing was allowed, patients came to a therapy center for a first assessment. Exclusion criteria were: additional injury apart from the ankle fracture, other musculoskeletal disorder, neurological problems, any condition that prevents the patients from performing a gait analysis test or complete self-assessment questionnaires. Out of the ninety-two patients, 41 participated. Main reason for not entering the study was that patients did not want to commit to a long-term follow-up study. Other reasons were pregnancy, vision problem, recruiting military service, and seeking other medical intervention.

Seventy-two healthy volunteers served as controls. This group was part of a larger database of healthy individuals that was collected by the current study researchers, at the study’s treatment site which is a private therapy center. Patients were healthy employees, caregivers and family members. This group was matched for age, gender and body mass index (BMI) and included healthy people without a history of any musculoskeletal problems and neurological problems.

All patients were informed of the study procedure including its purpose, protocol and any known risks and were asked to sign a consent form that was approved by the ethics committee.

### Gait analysis

A computerized mat was used to measure spatiotemporal gait parameters (GAITRite® system, CIR Systems Inc. Peekskill, NY, USA) [22,23]. During the gait test, all patients walked barefoot at a self-selected speed. Patients walked 3 meters before and after the walkway mat to allow sufficient acceleration and deceleration time outside the measurement area. Each gait test included 6 walks and the mean value of the 6 walks was calculated for each of the following parameters: velocity (m/s); step length (cm); single limb support (SLS) phase (% Gait cycle).

Temporal distance (T-D) symmetry was calculated for SLS and step length using the formula:

$$ \frac{\mathrm{involved}\kern0.5em \hbox{-} \kern0.5em \mathrm{uninvolved}}{\left(\mathrm{involved}\kern0.5em +\kern0.5em \mathrm{uninvolved}\right)/2}\times 100 $$

A symmetry index value of zero represents perfect symmetry and up to 5% difference between limbs was considered normal [24].

### Questionnaires

Patients were asked to complete two self-assessment questionnaires. The Foot and Ankle Outcome Score (FAOS) was used to evaluate patients symptoms [25]. This questionnaire comprises 42 Likert scale questions. Five sub-categories are calculated including symptoms (7 questions), pain (9 questions), function (17 questions), sport performance (5 questions) and quality of life (4 questions). Results range from 0 to 100. A score of 0 indicates a poor score and a score of 100 indicates a best score.

The Short Form (SF)-36 Health Survey (SF-36) was used to evaluate quality of life [26]. Posner et al. have concluded that the SF-36 is a useful tool to assess outcomes post ankle fracture [27]. The SF-36 is scored between 0 and 100, with 0 indicating the worst quality of life and 100 indicating the best quality of life. The questionnaire contains 36 questions of which 8 sub-categories are calculated: physical functioning, role-physical, bodily pain, general health, vitality, social functioning, role-emotional, mental health. Furthermore, 2 summarizing scores, physical health score (PHC) and a mental health score (MHS) are also calculated.

The American Orthopaedic Foot and Ankle Score (AOFAS) was used to evaluate the clinical condition of the patients [28]. This questionnaire was completed by trained physiotherapist. The AOFAS is scored between 0 and 100, with 0 indicating worst clinical condition and 100 indicating best clinical condition.

### Functional test

All patients performed the 6-min walk test which is a valid test that evaluates the functional status of the patient [29]. Patient is asked to walk the longest possible distance during 6 minutes and the total distance is measured. A low score indicate a short walking distance (i.e. worse functional status), whereas a high score indicate longer walking distance (i.e. better functional status).

### Data collection

All Ankle fracture patients came to a private therapy center and underwent the following assessments: the medical records of the patients were scanned and saved in the patient’s file; anthropometric measures of height, weight and age were captured. Each patient was asked to perform a gait analysis test and to complete the two self-assessment questionnaires. A trained physiotherapist completed the AOFAS and measured the ankle joint range of motion (ROM) in the sagittal (plantar flexion/dorsiflexion) and coronal plane (inversion/eversion). In addition, patients were asked to complete the 6-min walk test. All patients were instructed to refrain from taking pain medications, including paracetamol and non-steroidal anti-inflammatory drugs, for a period of 3 days prior to the clinical and gait evaluation.

The healthy population had only spatiotemporal gait analysis and SF-36 self-assessment questionnaire.

### Statistical analysis

Data were analyzed with IBM SPSS software version 21.0 and were presented as frequencies and percentages for categorical variables and as mean and standard deviation for all gait spatiotemporal parameters and self-evaluation questionnaires, followed by 95% confidence interval for the mean. Non-parametric one-sample Kolmogorov-Smirnov tests were calculated to compare the observed cumulative distribution function for the continuous variables with the Normal theoretical distribution. To demonstrate the differences in spatiotemporal gait parameters and self-evaluation questionnaires within the study groups, one-way ANOVA tests were performed. Once significant differences between groups’ means were determined, post hoc tests and pairwise multiple comparisons (Bonferroni test) were performed to determine which means differ.

## Results

Forty-one patients with an ankle fracture met inclusion criteria. There mean age (sd) was 47.3 (14.6) years. 12 patients (29%) had unimalleolar fracture, 15 patients (37%) had bimalleolar fracture and 14 patients (34%) had trimalleolar fracture. Furthermore, patients were also classified with deltoid ligament injury, syndesmosis injury or both. In the unimalleolar fracture group none of the patients had both a deltoid ligament injury and a syndesmosis injury, none had a deltoid ligament injury, and 4 patients (33%) had syndesmosis damage. In the bimalleolar fracture group one patient (6%) had both a deltoid ligament and syndesmosis damage, 4 patients (26%) had syndesmosis damage and none of the patients had solely a deltoid ligament injury. In the trimalleolar fracture group two patients (14%) had both a deltoid ligament and syndesmosis damage, 6 patients (42%) had syndesmosis damage and none of the patients had solely a deltoid ligament injury. There were no significant differences between groups in regard to prevalence of soft tissue injury (*p* = 0.328, *p* = 0.640 and *p* = 0.654 for deltoid ligament injury, syndesmosis injury or a combination of both, respectively). In an additional analysis we examined the differences in all measured variables between patients with syndesmosis injury and patients without syndesmosis injury and another comparison between patients with deltoid ligament injury and patients without and did not find significant differences between groups.

There were no significant differences between ankle fracture severity groups in regard to age, gender distribution and BMI. Furthermore, there were no significant differences between ankle severity groups in the time frame between injury and first assessment. Mean time (sd) of assessment for the unimalleolar, bimalleolar and trimalleolar groups were 66.1 (28.2), 68.1 (15.6) and 64.5 (17.4) days, respectively. Seventy-two healthy people matched for age, BMI and gender distribution served as controls. There mean (sd) age was 47.1 (17.7) years. Patients’ characteristics are summarized in Table 1.

**Table 1:** **Patients characteristics**

	**Unimalleolar**	**Bimalleolar**	**Trimalleolar**	**Healthy controls**	***p***
N (F/M)	12 (5/7)	15 (10/5)	14 (9/5)	72 (36/36)	0.437
Age (years)	50.3 (13.2)	47.9 (16.3)	44.1 (14.2)	47.1 (17.7)	0.815
Height	171.5 (7.2)	165.0 (6.2)	169.8 (9.5)	168.6 (9.4)	0.335
Weight	79.8 (16.8)	71.6 (13.2)	80.3 (15.7)	73.1 (14.1)	0.199
BMI (kg/m^2^)	27.0 (4.5)	26.7 (5.0)	28.1 (6.3)	25.5 (3.4)	0.185
Time from injury (days)	70.5 (26.0)	68.1 (15.6)	64.5 (17.4)	-	0.812

There were no significant differences between ankle fracture groups in ankle ROM in the sagittal plane and in the frontal plane, except for plantarflexion angle of the operated leg. Mean (sd) dorsiflexion angle of the operated leg was 0.8 (6.7) degrees, −3.3 (6.2) degrees and −0.8 (7.6) degrees for the unimalleolar, bimalleolar and trimalleolar groups respectively (*p* = 0.365). Mean (sd) plantarflexion angle of the operated leg was 46.7 (5.8) degrees, 44.6 (8.4) degrees and 40.6 (7.5) degrees for the unimalleolar, bimalleolar and trimalleolar groups respectively (*p* = 0.014). Mean (sd) inversion angle of the operated leg was 8.8 (3.8) degrees, 6.7 (3.7) degrees and 5.6 (3.6) degrees for the unimalleolar, bimalleolar and trimalleolar groups respectively (*p* = 0.058). Mean (sd) eversion angle of the operated leg was −0.4 (3.3) degrees, 0.0 (6.0) degrees and 2.5 (4.0) degrees for the unimalleolar, bimalleolar and trimalleolar groups respectively (0.893).

### Gait analysis

Significant differences were found between groups in all gait parameters including gait velocity, involved and uninvolved step length, involved and uninvolved SLS. All three fracture severity groups were significantly below the normal range. Patients with unimalleolar ankle fracture walked significantly faster compared to bimalleolar fracture (*p* = .016), but not compared to trimalleolar fracture (*p* = .239). They also had a significant longer step length in the uninvolved leg compared to the bimalleolar and trimalleolar groups (*p* = .002 and *p* = .041, respectively). Finally, the unimalleolar ankle fracture group had significant higher SLS values in the involved leg compared to both the bimalleolar group and trimalleolar group (*p* = .002 and *p* = .009, respectively). There were no significant differences in all gait parameters between the bimalleolar ankle fracture group and the trimalleolar ankle fracture group. Table 2 summarizes the differences in gait patterns between groups.

**Table 2:** **Differences in gait patterns between ankle fracture groups and controls**

	**Unimalleolar**	**Bimalleolar**	**Trimalleolar**	**Healthy controls**	***p***
Velocity (cm/s)	74.8 (29.0)	48.2 (21.6)	56.7 (23.8)	118.0 (20.2)	*p* < 0.001
	[56.4-93.2]	[36.2-60.1]	[42.9-70.4]	[113.3-122.8]	
Involved SL (cm)	61.6 (11.5)	41.6 (12.9)	48.1 (13.5)	62.9 (8.0)	*p* < 0.001
	[44.3-59.0]	[34.5-48.8]	[40.3-55.9]	[61.1-64.8]	
Uninvolved SL (cm)	43.0 (14.7)	26.2 (12.4)	30.4 (13.9)	62.8 (8.3)	*p* < 0.001
	[33.6-52.3]	[19.4-33.1]	[22.4-38.5]	[60.9-64.8]	
Involved SLS (% GC)	29.6 (6.8)	21.6 (6.2)	22.5 (7.6)	39.9 (1.5)	*p* < 0.001
	[25.3-33.9]	[18.2-25.0]	[18.1-26.9]	[39.5-40.2]	
Uninvolved SLS (% GC)	33.9 (5.7)	37.5 (5.5)	36.9 (7.2)	39.9 (1.5)	*p* < 0.001
	[30.3-37.5]	[34.5-40.5]	[32.8-41.0]	[39.6-40.3]	

*Abbreviations:* *SL* step length, *SLS* single limb support, *GC* gait cycle. Significance was set to *p* < 0.05.

An additional analysis of the gait patterns examined limb symmetry. Significant asymmetry was found in step length and SLS in all fracture groups but not in healthy controls. Step length asymmetry was 162% (*p* = .002), 119% (*p* = .003), and 131% (*p* = .001) for unimalleolar, bimalleolar and trimalleolar respectively. SLS asymmetry was 63% (*p* = .004), 256% (*p* = .001) and 189% (*p* = .001) for unimalleolar, bimalleolar and trimalleolar, respectively.

### Questionnaires

**Figure 1:**
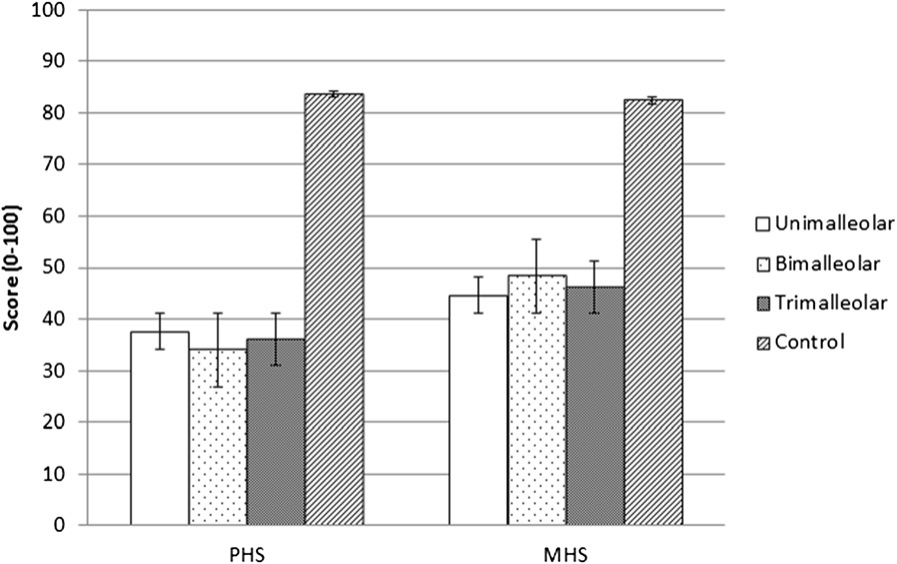
**SF-36 scores in the 3 ankle fracture groups and controls.** Abbreviation: PHS-Physical Health Score; MHS-Mental Health Score. Significant values were found between health controls and all 3 ankle fracture groups. There were no significant differences between the ankle fracture groups. *p*-value was set to *p* < 0.05.

SF-36 health survey scores were significantly different between groups. Significant differences were found between all 3 ankle fracture groups and healthy controls in all 8 sub-scale categories and in the summerising scores (PHS and MHS). There were no significant differences between ankle fracture groups in all SF-36 sub-scale categories. Figure 1 illustrates the differences between ankle fracture groups and healthy controls.

The AOFAS clinical assessment form was statistically different between groups (*p* = .017). Patients with unimalleolar fracture had a mean ± sd score of 59.0 ± 18.0, patients with bimalleolar fracture had a mean ± sd score of 37.7 ± 15.4 and patients with trimalleolar fracture had a mean ± sd score of 40.4 ± 20.0. Significant differences were found between the unimalleolar fracture group and the bimalleolar and trimalleolar fracture groups (*p* = .011 and *p* = .034, respectively). There were no significant differences between the bimalleolar fracture group and the trimalleolar fracture group (*p* =1.000).

There were no significant differences between fracture groups in FAOS total score and its 5 sub-scale categories.

### Functional test

Significant differences were found between ankle fracture groups in the 6-min walk test (*p* = .024). As fracture severity increased the walking distance of the patient decreased. Patients with unimalleolar fracture walked a mean ± sd distance of 376.3 ± 136.0 m, patients with bimalleolar fracture walked a mean ± sd distance of 250.3 ± 145.4 m and patients with trimalleolar fracture walked a mean ± sd distance of 201.2 ± 179.1. Significant differences in walking distance were found between patients with unimalleolar fracture and patients with trimalleolar fracture (*p* = .020). There were no significant differences between patients with unimalleolar fracture and patients with bimalleolar fracture (*p* = .128), and between patients with bimalleolar fracture and patients with trimalleolar fracture (*p* =1.000).

## Discussion

The functional condition of patients following ankle fracture has been well examined, but results remain unclear as researchers reported contradicting findings [8–11]. A long-term follow-up study of patients following unimalleolar and bimalleolar ankle fracture found that more than half of the patients still report pain, stiffness and swelling, and almost half of them had functional disabilities [30]. Although age was a dominant discriminator, fracture severity type should also be considered. A previous study has evaluated the gait patterns and symmetry in patients following ankle fracture, however no significant differences were found between fracture severity groups [16]. They evaluated only young population and months post-surgery. A two-year follow-up study on the functional outcomes and quality of life of patients with type B ankle fracture showed that nearly 60% of the patients had good clinical outcomes, however patients’ sensation of full recovery was reported by only 37%, 40% had work-related problems and 60% complained of ankle related problems with leisure or sports activities. Furthermore, they found that quality of life was negatively affected in patients with ankle fractures two-year after the injury [27]. The current study characterized the gait patterns and clinical symptoms of patients following ankle fracture compared to controls and evaluated the differences between ankle fracture severity groups (unimalleolar, bimalleolar and trimalleolar fracture). We found that all ankle fracture groups had a compromised gait pattern and poorer quality of life compared to healthy controls. Significant differences were also found within the ankle fracture groups in most parameters, especially between patients with unimalleolar fracture compared to bimalleolar and trimalleolar fracture.

Patients with an ankle fracture injury, which were treated surgically with immobilization period, presented compromised gait pattern and clinical symptoms. This is not surprising as this group of participants was examined immediately once weight-bearing activities were approved (2–3 months post ankle injury). We aimed to characterize the differences between ankle fracture severity groups early in the rehabilitation process and hypothesized that there will be a linear correlation between fracture severity group and functional disability (i.e. patients with unimalleolar will present better results compared to bimalleolar fracture and trimalleolar fracture and that patients with bimalleolar fracture will present better results compared to trimalleolar fracture). Our hypothesis was only partially supported with the study results. Regarding their gait pattern, patients with unimalleolar fracture presented better gait results compared to patients with bimalleolar and trimalleolar fracture in most gait parameters. But, patients with bimalleolar fracture were not statistically different compared to trimalleolar fracture patients. Interestingly, patients with bimalleolar fracture presented a slightly worse gait pattern compared to trimalleolar fracture patients. Furthermore, patients with unimalleolar fracture had better clinical score and functional score compared to bimalleolar fracture and trimalleolar fracture (AFAS, 6-min walk test). Similar to the gait results, there were no significant differences between the bimalleolar fracture group and trimalleolar fracture. Overall it seems that patients with unimalleolar fracture are at a better functional condition immediately with weight-bearing permission compared to bimalleolar and trimalleolar fracture. This was determined with both objective and subjective measures in order to make this characterization sound and valid. Moreover, it seems that there are no significant differences between patients with bimalleolar fracture and patients with trimalleolar fracture. Based on the current study results it may be postulated that unimalleolar fracture is a different injury than bimalleolar and trimalleolar fracture and can be considered as a mild injury. Furthermore, it may be postulated that both bimalleolar fracture and trimalleolar fracture affect the functional condition of the patient to the same extend and should be considered as equal. It is important to note that there were no significant differences between ankle fracture groups in the prevalence of soft tissue injuries. Furthermore, there were no significant differences between patients with a soft tissue injury and patients without a soft tissue injury in all measured variables. Based on these findings it may be assumed that the presence of a soft tissue injury is not a covariate to the results presented above. Our results partially support previous results that have reported no significant differences in functional outcomes between fracture severity classifications [8–10]. In contrast, our results also support the results of Tejwani et al. who also concluded that the functional outcome for patients with a bimalleolar fracture is worse than that for patients with a unimalleolar fracture [31]. They did not, however, include a group of patients with trimalleolar fracture. Future research should examine whether this trend changes in the long-term. It could be that fracture severity will have a long-term effect and that patients with trimalleolar fracture will have a slower rehabilitation period compared to patients with bimalleolar fracture. Furthermore, future research may also use these measures to evaluate intervention outcomes.

This study had some limitations. First, this study was applied to a relatively small sample groups. Although the study had strong power to detect differences between the ankle fracture groups and the control group, it had lower statistical power for comparing the ankle fracture groups within themselves. Nevertheless, it seems that even with a sample size of 60 patients in each group (bimalleolar and trimalleolar), there is not enough power to detect statistical significant differences (the power is below 80%). This implies that the fact that there were no significant differences between bimalleolar fracture and trimalleolar fracture groups is not due to small sample size, but rather a true reflection of these groups. Future research should examine the changes in gait patterns and clinical symptoms in larger cohort groups. Secondly, only spatiotemporal gait analysis was applied. Evaluating gait differences in a comprehensive 3 dimensional gait analysis could have added additional and wider information regarding the changes in gait patterns between the ankle fracture groups. Nevertheless, we sought of using a simple, objective measuring tool that can be implemented in any clinic with immediate results. Thirdly, this was a one-session evaluation of patients immediately following ankle fracture. Future research should examine long-term differences in gait patterns and clinical outcomes between severity groups.

## Conclusions

Patients with ankle fracture present altered gait patterns and clinical symptoms compared to healthy controls. Patients with unimalleolar fracture present significant better results compared to bimalleolar and trimalleolar ankle fracture. There were no significant differences between bimalleolar fracture and trimalleolar fracture patients immediately post injury with weight-bearing approval. Such characterization was done while using both objective and subjective measures. Using these parameters may also serve as tools to evaluate treatment outcomes. Furthermore, based on the results of the present study it may be suggested that although rehabilitation following ankle fracture is essential for all types of fracture severity, they should be personally fitted. Bimalleolar and trimalleolar fracture groups may need a more extensive rehabilitation program as they present lower scores compared to unimalleolar patients.

## Abbreviations

BMI: Body mass index
SLS: Single limb support
FAOS: The Foot and Ankle Outcome Score
PHC: Physical health score
MHS: Mental health score
AOFAS: The American Orthopaedic Foot and Ankle Score
ROM: Range of motion
